# Kynurenine Pathway Activation in Human African Trypanosomiasis

**DOI:** 10.1093/infdis/jiw623

**Published:** 2016-12-24

**Authors:** Jeremy M. Sternberg, Caroline M. Forrest, R. Neil Dalton, Charles Turner, Jean Rodgers, Trevor W. Stone, Peter G. E. Kennedy

**Affiliations:** 1 Institute of Biological and Environmental Sciences, University of Aberdeen,; 2 Institute of Neuroscience and Psychology,; 3 Institute of Biodiversity, Animal Health, and Comparative Medicine,; 4 Institute of Neuroscience and Psychology, and; 5 Department of Neurology, College of Medical, Veterinary, and Life Sciences, University of Glasgow, and; 6 WellChild Laboratory, Evelina London Children’s Hospital, King’s College London, United Kingdom

**Keywords:** African trypanosomiasis, meningoencephalitic, neuroinflammation, kynurenine, tryptophan.

## Abstract

**Background.:**

The kynurenine pathway of tryptophan oxidation is associated with central nervous system (CNS) inflammatory pathways. Inhibition of this pathway ameliorates CNS inflammation in rodent models of the late (meningoencephalitic) stage of human African trypanosomiasis (HAT). In this study, we evaluate whether the kynurenine pathway is activated in clinical HAT and associated with CNS inflammatory responses.

**Methods.:**

We measured cerebrospinal fluid (CSF) tryptophan and kynurenine metabolite concentrations in patients infected with *Trypanosoma brucei rhodesiense*, using liquid chromatography–mass spectrometry.

**Results.:**

Kynurenine concentration in CSF was increased in both the early and late stages of disease, with a progressive increase in tryptophan oxidation associated with stage progression. Kynurenine pathway activation was associated with increases in neuroinflammatory markers, but there was no clear relationship to neurological symptoms.

**Conclusions.:**

CNS kynurenine pathway activation occurs during HAT, including cases prior to the current diagnostic cutoff for late-stage infection, providing evidence for early CNS involvement in HAT. Metabolite data demonstrate that the kynurenine-3-monooxygenase and kynurenine aminotransferase branches of the kynurenine pathway are active. The association between tryptophan oxidation and CNS inflammatory responses as measured by CSF interleukin 6 (IL-6) concentration supports a role of kynurenine metabolites in the inflammatory pathogenesis of late-stage HAT.

African trypanosomiasis (sleeping sickness) is caused by the tsetse fly–transmitted protozoan parasites *Trypanosoma brucei rhodesiense* and *Trypanosoma brucei gambiense*. Infection progresses through an initial hemolymphatic stage and culminates in the meningoencephalitic stage, during which the invasion of the central nervous system (CNS) by trypanosomes leads to neurological disturbances, neuroinflammatory pathology, and, if untreated, death [1]. Treatment of the late stage of the disease is hampered by a posttreatment reactive encephalopathy (PTRE) that is associated with an exacerbation of the neuroinflammatory response [2]. This is particularly severe following the use of melarsoprol, a trivalent arsenical drug, which is presently the only treatment for meningoencephalitic *T. b. rhodesiense* infection. The neuroinflammatory reaction plays a central role in the pathogenesis of the disease and the adverse reactions to treatment. An improved understanding of the mechanisms directing these processes may lead to modifications of the treatment strategy that would limit the extent of the PTRE. Indeed, both nonsteroidal antiinflammatory drugs and counterinflammatory cytokine treatments have been demonstrated to reduce neuropathology in animal models of meningoencephalitic human African trypanosomiasis (HAT) infections [3, 4].

The kynurenine pathway is the main pathway of tryptophan degradation. An outline of the key enzymes and pathway metabolites is provided in Supplementary Figure S1 for reference. Kynurenine pathway metabolites have a range of activities in the CNS, including neuroprotective, neurotoxic, and immunosuppressive functions, and the pathway is activated in association with inflammatory pathways in the CNS in both infectious diseases [5–7] and neurodegenerative conditions [8–10]. Depletion of the first compound on the pathway (tryptophan), together with the actions of kynurenine metabolites on immune cell function [11–13], may lead to limitations on the proliferation of both microbial pathogens and immune cells in the relevant tissues. Evidence for a role of kynurenine pathway metabolites in the pathogenesis of meningoencephalitic HAT was recently obtained in a mouse model [14]. Administration of 3,4-dimethoxy-N-[4-(3-nitrophenyl)thiazol-2-yl]benzenesulfonamide (Ro-61–8048; [15]), an inhibitor of kynurenine-3-monooxygenase (KMO), in that model led to a reduction in neuroinflammatory response, suggesting that such inhibitors may offer potential therapeutic tools to ameliorate PTRE in clinical cases. Since this pathway is enmeshed in the CNS inflammatory response, it is also possible that kynurenine metabolites might also act as biomarkers for meningoencephalitic disease, for which there is an urgent need to develop more-precise stage diagnostic criteria [16].

In this study, we use clinical samples of CSF from patients with HAT to establish whether kynurenine pathway activation occurs in the human condition. We assess whether kynurenine pathway metabolites are associated with either neuroinflammatory markers or neurological sequelae and evaluate their potential as stage diagnostic markers.

## PATIENTS AND METHODS

This was a retrospective study involving cerebrospinal fluid samples from 40 patients with HAT and 10 uninfected individuals. The samples in this study were collected under protocols approved by ethics committees in Uganda (Uganda National Council for Science and Technology) and the United Kingdom (North of Scotland Research Ethics Committee), conforming to the principles of the Declaration of Helsinki. Ethical consent forms were designed in English and translated into local languages. Consent was given as a signature or a thumbprint after verbal explanation. For those aged <18 years, consent was given by the legal guardian.

A total of 40 patients with HAT were recruited at Lwala Hospital, Kaberamaido District and Serere Health Center (Serere District, Eastern Region) between November 2008 and March 2010. Full details of the recruitment protocols, treatment regimens, disease progression characteristics, and clinical examination methods have been published elsewhere [17]. Patients with intercurrent malaria, filariasis, or schistosomiasis were excluded. Cerebrospinal fluid (CSF) samples were collected from all patients as part of normal diagnostic and staging procedures. Staging was performed in accordance with World Health Organization criteria [18], in which late-stage infection was defined by the presence of parasites in the lumbar CSF and/or a CSF white blood cell count (WBC) of >5 cells/µL. A further 10 control CSF samples were obtained from patients with suspected HAT who were subsequently confirmed as uninfected. Aliquots of 1–2 mL of CSF were centrifuged (at 450 × *g* for 10 minutes at 4°C), and the supernatant was frozen and then maintained in liquid nitrogen until transfer to the United Kingdom. After transport via airfreight (involving storage for 24 hours on dry ice), samples were maintained at −80°C until analysis.

### Kynurenine Pathway Metabolite Analysis

#### Liquid Chromatography–Tandem Mass Spectrometry

Tryptophan, kynurenine, and kynurenine metabolites were measured by liquid chromatography–tandem mass spectrometry, using a CTC PAL HTS-XT, Agilent 1260 Infinity liquid chromatograph and an AB SCIEX 6500 QTRAP mass spectrometer (AB Sciex UK, Warrington, United Kingdom). All results were calculated in Analyst 1.6.

#### Analytical Chemicals and Reagents

Tryptophan, kynurenine, kynurenic acid, anthranilic acid, 3-hydroxykynurenine, 3-hydroxyanthranilic acid, xanthurenic acid, picolinic acid, and quinolinic acid were obtained from Sigma Aldrich (Poole, United Kingdom). The stable isotope–labeled internal standards were ^2^H_5_-tryptophan (QMx Laboratories, Thaxted, United Kingdom), ^2^H_6_-kynurenine sulfate and ^2^H_5_-kynurenic acid (CK isotopes, Ibstock, United Kingdom), and ^2^H_3_-picolinic acid and ^13^C_3_,^15^N-quinolinic acid (LGC standards, Teddington, United Kingdom). Acetonitrile and methanol were obtained from Rathburn Chemicals (Walkerburn, United Kingdom) and Fisher Scientific UK (Loughborough, United Kingdom), respectively.

Standards of each analyte were used to automatically tune in multiple reaction monitoring (MRM) mode. Transition optimizations were performed manually. Xanthurenic acid was significantly more sensitive in negative ion mode, but, with the chromatographic conditions used, there was massive matrix ion suppression. Consequently, xanthurenic acid was measured in positive ion mode.

Because analyte retention times on Chirobiotic-T columns tend to be matrix dependent, there is a critical requirement for the appropriate stable isotope internal standards. Consequently, when stable isotopes were unavailable, 2 separate transitions, when possible, were used to calculate and then confirm the result.

#### CSF Sample Preparation

For measurement of all analytes, 60 µL of blank (deionized water), aqueous standards, and CSF were mixed with 10 mL of the stable isotope mix (20 µL of 10 mM ^2^H_5_-tryptophan; 2 µL of 16 mM ^2^H_6_-kynurenine sulfate; 2 µL of 2 mM ^2^H_5_-kynurenic acid; 5 µL of 15 mM ^13^C_3_,^15^N-quinolinic acid; and 5 µL of 40 mM ^2^H_3_-picolinic acid) in 75 µL of methanol, mixed by vortexing, and centrifuged for 5 minutes at 20 817 × *g*. Supernatants were transferred to a 96-well deep well polypropylene plate, a sealing mat was applied, and the plate was placed in the CTC autosampler and cooled to 7.5°C, awaiting injection.

#### Positive Ion MRM Mode Acquisitions

Isocratic chromatography of tryptophan and kynurenine, kynurenic acid, anthranilic acid, 3-hydroxykynurenine, 3-hydroxyanthranilic acid, and xanthurenic acid (injection volume, 10 µL; data acquisition time, 7 minutes) was performed on a 5-µm Astec Chirobiotic-T column (10 cm [length] × 2.1 mm [internal diameter] guard column), using a 1:1 ratio of acetonitrile to water with 0.025% formic acid at a flow rate of 200 µL/minute. Tandem mass spectrometry parameters were as follows: curtain gas, 40; CAD gas, medium; ion source voltage, 5250 V; gas temperature, 400°C; gas 1, 25; and gas 2, 25.

#### Negative Ion MRM Mode Acquisitions

Isocratic chromatography of quinolinic acid and picolinic acid (injection volume, 10 µL; data acquisition time, 4 minutes) was performed on a 5-µm Astec Chirobiotic-T (10 cm [length] × 2.1 mm [internal diameter] guard column), using 62.5% water/acetonitrile at a flow rate of 225 µL/minute. Tandem mass spectrometry parameters were as follows: curtain gas, 45; CAD gas, medium; ion source voltage, −4500 V; gas temperature, 400°C; gas 1, 30; and gas 2, 30. Full details of mass spectrometry conditions are described by Forrest et al [19].

### CSF Cytokine Assays

Interferon γ (IFN-γ), interleukin 6 (IL-6), and interleukin 10 (IL-10) concentrations were measured using a solid-phase sandwich enzyme-linked immunosorbent assay (OptiEIA; BD Pharmingen), as described previously [20]. Biological limits of detection were 1.8, 8.3, and 1.6 pg/mL, respectively

### Statistical Analysis

CSF analyte data were left skewed and could not be consistently transformed. Therefore, nonparametric inferential statistical analyses (indicated in figure legends or the text) were performed using JMP10.0 (SAS, Cary, NC). For descriptive and inferential statistical analysis, results below the limit of detection were adjusted to 0.5 times the limit of detection. For analysis of diagnostic power, areas under the receiver operator curve (AUROCs) were calculated using the logistic modeling platform in JMP 10.0. Significance values in multiple correlation analyses were adjusted as described by Benjamini and Hochberg [21].

## RESULTS

### Patients With HAT and Controls

A summary of the parasitological and demographic characteristics and neurological assessment of the patients is presented in Table 1. Neurological symptoms (gait ataxia, somnolence, tremor, and abnormal Glasgow Coma Scale score) were predominantly observed in late-stage cases. Neurological assessments were not available for control subjects.

**Table 1.** Demographic and Symptomatic Characteristics of Uninfected Controls and Patients With Early Stage or Late Stage Human African Trypanosomiasis

Characteristic	Controls (n = 10)	Early Stage (n = 11)	Late Stage (n = 29)
Age, y, median (range)	30 (21–69)	22 (15–70)	22 (14–45)
Male sex; female sex	6; 4	3; 8	14; 15
CSF WBC count, cells/µL, median (range)	NA	3 (1–5)	25 (8–308)
Somnolence	NA	0/11	16/29^a^
Gait ataxia	NA	1/11	11/27^a,b^
Tremors	NA	1/11	11/29^a^
GCS score <15	NA	0/11	7/29

Abbreviations: CSF, cerebrospinal fluid; GCS, Glasgow Coma Scale; NA, not applicable; WBC, white blood cell.

^a^Significantly greater, compared with patients with early stage HAT (*P* < .05 by the χ^2^ test).

^b^Data were unavailable for 2 patients with late-stage HAT.

### Kynurenine Pathway Metabolite Concentrations in HAT Cases

In late-stage HAT cases, CSF tryptophan levels were significantly lower than those in either early stage cases or uninfected samples (Table 2), although the range of outlying concentrations in both early stage and late-stage cases demonstrates that the CSF tryptophan concentration is of limited stage diagnostic value (AUROC = 0.72). Conversely, the L-kynurenine concentration was significantly increased in all HAT cases (both early and late stage), compared with findings for uninfected individuals.

**Table 2.** Cerebrospinal Fluid Concentrations of Tryptophan, Kynurenine, and Downstream Metabolites in Uninfected Controls and Patients With Early Stage or Late-Stage Human African Trypanosomiasis

Variable	Concentration, nmol/L			
	Uninfected (n = 10)	Early Stage (n = 11)	Late Stage (n = 29)	*P*
Tryptophan	1003.5 (531.6–1195)	937 (430–1560)	335 (98.8–634)	<.05^a^
Kynurenine	112.5 (65.75–198)	330 (190–460)§	226 (148–729)	<.05^a^
Kynurenic acid	4.21(1.89–10.4)	7.37 (5.2–9.29)	6.54 (2.88–11)	
Anthranilic acid	1.35 (1.02–2.25)	1.81 (1.57–2.08)	1.73 (1.49–2.31)	
3-HK	10.3 (8.48–12.05)	15.2 (9.14–36.6)	15.4 (7.93–27.9)	
3-HAA	0.25 (0.25–0.64)	0.25 (0.25–0.48)	0.25 (0.25-0.25)	

Data are median values (interquartile ranges).

Abbreviations: 3-HAA, 3-hydroxyanthranilic acid; 3-HK, 3-hydroxykynurenine.

^a^By the Dunn multiple comparisons test, compared with uninfected controls.

**Figure 1.**
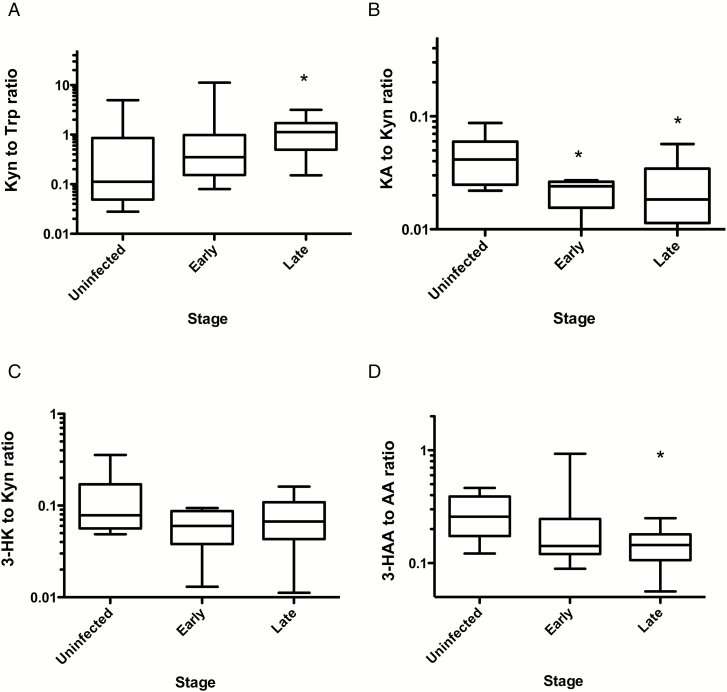
*A*, Ratios of cerebrospinal fluid concentrations, in nmol/L, of kynurenine (Kyn) to tryptophan (Trp; *A*), kynurenic acid (KA) to Kyn (*B*), 3-hydroxykynurenine (3-HK) to Kyn (*C*), and 3-hydroxyanthranilic acid (3-HAA) to anthranilic acid (AA; *D*) in 10 uninfected controls, 11 patients with early stage human African trypanosomiasis (HAT), and 29 patients with late-stage HAT. Boxes show median values and interquartile ranges. Whiskers indicate ranges from the 10th to 90th percentiles. *P < .05, by the Dunn multiple comparisons test, compared with uninfected controls.

The ratio of CSF kynurenine to tryptophan was calculated as an index of tryptophan oxidation via indoleamine-2,3-dioxygenase (IDO) and tryptophan-2,3-dioxygenase (TDO) activities and indicated significantly increased tryptophan oxidation in late-stage HAT cases (Figure 1A), although, as in the case of tryptophan, the stage diagnostic power of this parameter was weak (AUROC = 0.68).

Metabolites representing subsequent stages of the kynurenine pathway were also analyzed (an outline of the key enzymes and pathways is provided in Supplementary Figure 1). Although increases in 3-hydroxykynurenine concentration in patients with HAT (both early and late stage) suggested activation of the KMO branch of the pathway, this was not significant, probably because of the small size of the control group. We therefore examined the correlation coefficients of L-kynurenine metabolites within the infection cases as a means to identify the dominant kynurenine metabolic pathways in patients with HAT (Table 3). Significant correlations of kynurenine concentration with those of 3-hydroxykynurenine and kynurenic acid provides evidence of KMO and kynurenine aminotransferase (KAT) activity.

**Table 3.** Relationships of Cerebrospinal Fluid Kynurenine Concentration and Downstream Metabolites in Patients With Human African Trypanosomiasis

Variable	3-HK	*P*	Anthranilic Acid	*P*	3-HAA	*P*	Kynurenic Acid	*P*
Kynurenine	0.47	<.001	0.25	NS	0.11	NS	0.56	<.001
3-HK	…	…	0.28	NS	0.08	NS	0.13	NS
Anthranilic acid	…	…	…	…	0.03	NS	0.33	NS
3-HAA	…	…	…	…	…	…	0.05	NS

Data are Spearman rank correlation coefficients. Significance was corrected using the Benjamini-Hochberg method for multiple-test false-discovery rates.

Abbreviations: NS, not significant; 3-HAA, 3-hydroxyanthranilic acid; 3-HK, 3-hydroxykynurenine.

Modulation of KMO and KAT with infection stage was assessed by analysis of the product to substrate ratios. Kynurenic acid to kynurenine ratios were significantly reduced, compared with control values, in early stage and late-stage HAT cases (Figure 1B), indicating inhibition of the KAT branch of the kynurenine pathway. On the other hand, there was no significant change in 3-hydroxykynurenine to kynurenine ratios between infected and control subjects (Figure 1C), suggesting that the KMO branch of the pathway was predominant.

We were unable to measure the terminal metabolites—xanthurenic acid, quinolinic acid, and picolinic acid—that have previously been detected by this tandem mass spectrometry method in brain samples [19]. CSF levels of xanthurenic acid were <0.5 nmol/L, and accurate quantification would require the concentration of larger sample volumes prior to analysis.. The measurement of CSF levels of quinolinic acid and picolinic acid proved problematic owing to the small sample volumes available and very high ion suppression.

The ratio of 3-hydroxy-anthranilic acid to anthranilic acid was significantly reduced in late-stage HAT cases as compared to controls (Figure 1D), consistent with inflammatory activation [22], although because of the large variance in early stage cases this parameter is of no stage diagnostic value.

### Kynurenine Pathway and CNS Immune Activation

Stage progression in HAT was associated with significant increases in CSF levels of IL-6, IL-10, and IFN-γ (Table 4). Because the kynurenine to tryptophan ratio was also elevated in late-stage cases, we investigated whether the kynurenine pathway was associated with CNS immune activation, as measured by CSF cytokine concentration and white blood cell counts. There were strong and highly significant correlations between kynurenine to tryptophan ratios and CSF white blood cell count, IL-6 concentration, and IL-10 concentration (Table 5). The ratio of 3-hydroxy-anthranilic acid to anthranilic acid, which is inversely associated with neuroinflammatory activation in other pathologies [22], also exhibited a significant negative correlation with CSF IL-6 concentration (Table 5).

**Table 4.** Cerebrospinal Fluid Concentration of Cytokines, by Human African Trypanosomiasis Stage

Cytokine	Concentration, by Disease Stage, pg/mL		
	Early (n = 11)	Late (n = 29)	*P* ^a^
IL-6	4.2 (4.2–6.0)	56.1 (4.2–222.1)	<.005
IL-10	9.8 (0.8–78.5)	326.4 (106.1–454.1)	<.005
IFN-γ	0.9 (0.9–12.9)	22.0 (0.9–38.4)	<.05

Data are median values (interquartile ranges).

Abbreviations: IFN-γ, interferon γ; IL-6, interleukin 6; IL-10, interleukin 10.

^a^By the Mann–Whitney test, compared with patients with early stage disease.

**Table 5.** Correlations of the Ratio of Kynurenine (Kyn) to Tryptophan (Trp) and the Ratio of 3-Hydroxyanthranilic Acid (3-HAA) to Anthranilic Acid (AA) to Cerebrospinal Fluid Markers of Immune Activation

Variable	3-HAA to AA Ratio	*P*	WBC Count	*P*	IL-6 Concentration	*P*	IL-10 Concentration	*P*	IFN-γ Concentration	*P*
Kyn to Trp ratio	0.19	NS	0.38	<.03	0.47	<.03	0.56	<.01	0.26	NS
3-HAA to AA ratio	…		0.12		−0.44	<.03	−0.25	NS	−0.05	NS
WBC count	…		…		0.33	NS	0.55	<.01	0.51	<.02
IL-6 concentration	…		…		…		0.73	<.001	0.35	NS
IL-10 concentration	…		…		…		…		0.61	<.01

Data are Spearman rank correlation coefficients. Significance was corrected using the Benjamini-Hochberg method for multiple-test false-discovery rates.

Abbreviations: IFN-γ, interferon γ; IL-6, interleukin 6; IL-10, interleukin 10; NS, not significant; WBC, white blood cell.

Because the KMO inhibitor Ro-61–8048 ameliorated neuroinflammatory responses in a mouse model of HAT [14], we investigated whether the primary metabolic product of this enzyme, 3-hydroxykynurenine, would exhibit a positive correlation with markers of neuroinflammation. However, no significant relationships were observed with the IL-6 level (Spearman ρ = 0.19), the IFN-γ level (ρ = −0.11), or CSF WBC pleocytosis (ρ = −0.04).

### Kynurenine Pathway and Neurological Symptoms

All patients were subject to neurological assessment on entering the study. There was no association between the concentration of any of the kynurenine metabolites and presentation with tremor, gait ataxias, or evidence of coma, as measured by a Glasgow Coma Scale score of <15. In somnolent patients, we did observe increased tryptophan oxidation activity (median kynurenine to tryptophan ratio, 1.40 in somnolent patients and 0.52 in nonsomnolent patients; *P* < .05 by the Mann–Whitney *U* test). Because somnolence was strongly associated with late stage cases (Table 1), we examined tryptophan oxidation in late-stage cases only. In this analysis, the apparent difference in kynurenine to tryptophan ratios between somnolent cases and nonsomnolent cases (median, 1.39 and 0.68, respectively) was not significant (*P* = .08).

## DISCUSSION

In this study, the concentrations of tryptophan and metabolites of the kynurenine pathway were measured in relation to disease status and CNS inflammatory responses in patients with HAT. There were increases in kynurenine concentration in the CSF during early stage and late-stage disease, with an increased kynurenine to tryptophan ratio in late-stage disease, in which depletion of tryptophan was evident. The increased kynurenine concentration in the CSF of patients with early stage HAT provides evidence for upregulation of CNS tryptophan metabolism before the onset of the late stage as defined by current diagnostic criteria. This finding is consistent with similar results in an independent clinical study of intrathecal immunoglobulin synthesis in *T. b. rhodesiense* HAT [23]. When combined with recent data on early CNS involvement in rodent models of African trypanosomiasis [24], these findings question the biological relevance of current stage diagnostic criteria.

The correlation between kynurenine and 3-hydroxykynurenine levels and between kynurenine and kynurenic acid are consistent with activity along both the KMO and KAT branches of the kynurenine pathway. The reduced product/substrate concentrations observed in infected individuals as compared to controls for the KAT branch but not for the KMO branch of the pathway indicate that KMO is the predominant pathway activated during infection, but for both pathways there appears to be no change in the relative activities of the 2 enzymes during progression from early stage to late-stage HAT.

While the immediate metabolic product of KMO, 3-hydroxykynurenine, is known to have neurotoxic activity [25–29], it is unlikely to be involved in the neuropathology of HAT since there was no relationship between its concentration in the CSF and either inflammatory markers (white blood cell counts or cytokines) or neurological symptoms. It remains possible that quinolinic acid, a downstream metabolite of 3-hydroxy-kyurenine, could be involved, because it also has marked neurotoxicity via NMDA receptors and other mechanisms [30]. The ratio between 3-hydroxy-anthranilic acid and anthranilic acid is inversely related to the presence of inflammation [22] and, interestingly, was significantly reduced in late-stage CSF samples. In addition, this metabolite was negatively correlated to the inflammatory CSF marker IL-6.

CNS immune activation in HAT was apparent in increases in both inflammatory (IL-6 and IFN-γ) and counterinflammatory (IL-10) cytokines with stage progression, and this was also reflected in increased CSF white blood cell concentration. These increases have been described in previous studies of patients with HAT [31]. Our data demonstrate an association between tryptophan oxidation and CNS inflammatory activity, as reflected in IL-6 concentration. While it cannot be stated with certainty whether this results from increased activity of IDO in the CNS and immune system cells or of TDO in the liver, the former is more likely because it is induced and activated by IFN-γ, whereas TDO is regulated primarily by stress-induced corticosteroids. Interestingly, the ratio of 3-hydoxy-anthranilic acid to anthranilic acid, which has been shown to be negatively associated with inflammatory pathology in other conditions [22], also demonstrated a significant negative relationship with the CSF IL-6 concentration but not IL-10 concentration in patients with HAT and may represent the first discrimination of the inflammatory response in the brain from the counterinflammatory regulation by IL-10 [32]. Cytokine correlations with the kynurenine to tryptophan ratio have been demonstrated in other disorders, such as cancer [33], meningitis [34], and atherosclerosis [35]. Activation of IDO induces the production of IL-10 [36, 37], which is a well characterized CNS response in HAT, while IL-10 has in turn been reported to inhibit the IFN-γ–induced activation of IDO in neurons [38]. This may reflect a novel mechanism by which coordination of the kynurenine pathway and inflammatory/counterinflammaotry regulation influence neuropathogenesis in HAT [32].

Because the kynurenine pathway includes potent modulators of neuronal function, such as quinolinic acid as an agonist [39] and kynurenic acid as an antagonist [40, 41], at NMDA-sensitive glutamate receptors, the relationship may provide a potential explanation of some of the neurological symptoms of HAT. However, in this study only somnolence was associated with increased kynurenine pathway activation, and because this association was confounded with the increased activation in the late stage of disease, a larger scale clinical study is required to establish the significance of this relationship.

In previous work we demonstrated that the inhibition of KMO leads to an amelioration of neuropathology in a mouse model of HAT CNS disease [14]. Similarly, in a murine form of cerebral malaria, KMO inhibition prevented the death of the infected mice, prolonging the normal 7-day survival period to at least 21 days [5]. While the results presented here in patients with HAT indicate that the initial product of KMO, 3-hydroxykynurenine, is not related to either inflammatory pathology or disease symptoms, it is possible that downstream kynurenine pathway metabolites such as quinolinic acid [30] induce HAT neuropathology, and further work using experimental models is required to understand the mechanistic consequences of KMO inhibition and whether this offers a potential avenue in the search for novel treatments of this and other CNS conditions exhibiting neuroinflammatory pathologies.

In conclusion, this study indicates that the kynurenine pathway is integrally involved in the development of the neuroinflammatory response associated with clinical cases of African trypanosomiasis. Although it may not be possible to utilize these metabolites as biomarkers for disease staging, the study provides further evidence that the current clinical stage diagnostic criteria may not reflect clinically relevant pathological processes in the CNS. In a retrospective study such as this, causal relationships cannot be precisely defined, but further work in experimental models will offer the potential to understand the mechanistic relationships of the kynurenine pathway and HAT neuropathology and hence provide a route to novel treatment strategies that may minimize or eliminate the risk of a severe PTRE in these critically ill patients.

## Supplementary Data

Supplementary materials are available at *The Journal of Infectious Diseases* online. Consisting of data provided by the authors to benefit the reader, the posted materials are not copyedited and are the sole responsibility of the authors, so questions or comments should be addressed to the corresponding author.

## Supplementary Material

Supplementary_Figure_1

SUPPLEMENTARY_FIGURE_LEGENDS

## Notes

***Financial support.*** This work was supported by the Wellcome Trust (grants 082786 [to J. M. S.] and 094691 [to P. G. E. K.]).

***Potential conflicts of interest.*** All authors: No reported conflicts. All authors have submitted the ICMJE Form for Disclosure of Potential Conflicts of Interest. Conflicts that the editors consider relevant to the content of the manuscript have been disclosed.
