# Supplementary figures and images for: Kynurenine Pathway Activation in Human African Trypanosomiasis
Source: J Infect Dis. 2016 Dec 24;215(5):806–12. doi: 10.1093/infdis/jiw623 (PMC5388295; doi:10.1093/infdis/jiw623)

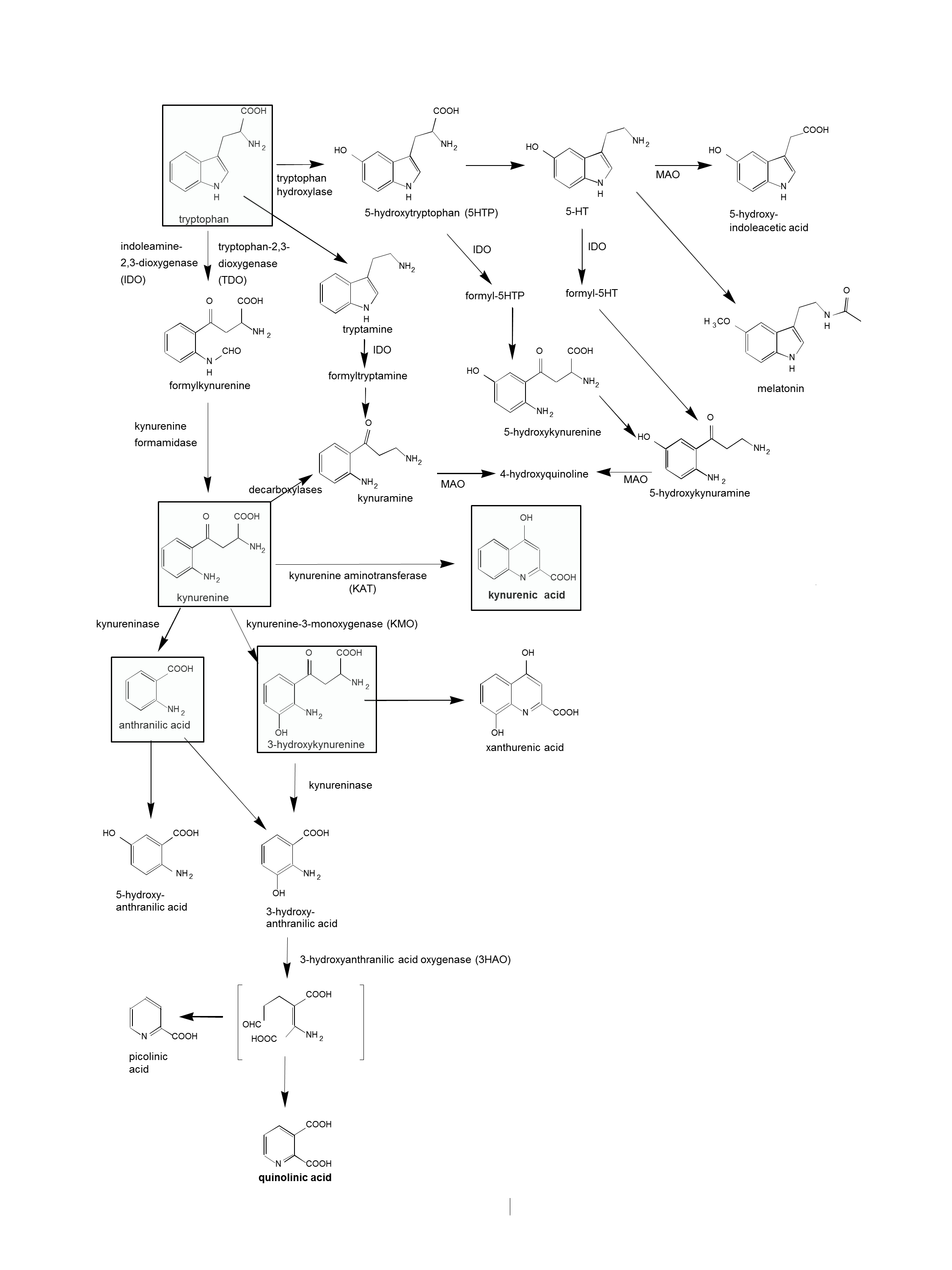

Supplement: Supplementary_Figure_1 [file jiw623_suppl_Supplementary_Figure_1.png]
