# Supplementary material for: Kynurenine Pathway Activation in Human African Trypanosomiasis
Source: J Infect Dis. 2016 Dec 24;215(5):806–12. doi: 10.1093/infdis/jiw623 (PMC5388295; doi:10.1093/infdis/jiw623)
Supplement: SUPPLEMENTARY_FIGURE_LEGENDS [file jiw623_suppl_SUPPLEMENTARY_FIGURE_LEGENDS.docx]

**SUPPLEMENTARY FIGURE LEGENDS:**

**Supplementary Figure 1:**

Outline of main components of tryptophan metabolism including kynurenine pathway. Boxes indicate the metabolites measured in this study.
